# A Synergistic Association Between Inflammation, Malnutrition, and Mortality in Patients With Diabetics

**DOI:** 10.3389/fnut.2022.872512

**Published:** 2022-06-02

**Authors:** Junjie Wang, Liling Chen, Zhidong Huang, Jin Lu, Yanfang Yang, Xiaoli Zhao, Jiabin Tu, Yuxiong Pan, Kunming Bao, Weihua Chen, Jiaming Xiu, Yong Liu, Longtian Chen, Shiqun Chen, Kaihong Chen

**Affiliations:** ^1^Department of Cardiology, Longyan First Affiliated Hospital of Fujian Medical University, Longyan, China; ^2^Department of Cardiology, Guangdong Provincial Key Laboratory of Coronary Heart Disease Prevention, Guangdong Cardiovascular Institute, Guangdong Provincial People's Hospital, Guangdong Academy of Medical Sciences, Guangzhou, China; ^3^Department of Cardiology, The Third Affiliated Hospital of Sun Yat-Sen University, Guangzhou, China; ^4^Department of Hematology, Longyan First Affiliated Hospital of Fujian Medical University, Longyan, China

**Keywords:** CONUT score, malnutrition, hs-CRP, all-cause mortality, inflammation, diabetes mellitus

## Abstract

**Background:**

Although inflammation is a known predictor for poor prognosis in patients with diabetics, few data report the synergistic association between inflammation, malnutrition, and mortality in patients with diabetics. We aim to explore whether malnutrition modifies the predictor of inflammation on prognosis.

**Methods:**

Nutritional status and inflammation were measured in 6,682 patients with diabetics undergoing coronary angiography or percutaneous coronary intervention between January 2007 to December 2018 from Cardiorenal Improvement Registry. Malnutrition was defined as Controlling Nutritional Status (CONUT) score, which was more than 1. High-sensitivity C-reactive protein (hs-CRP) exceeding the median was assessed as a high-risk inflammation. Cox regression models were used to estimate hazard ratios (HR) for mortality across combined hs-CRP and CONUT score categories.

**Results:**

During a median follow-up of 5.0 years (interquartile range: 3.0–7.6 years), 759 (11.36%) patients died. The mortality of the four groups (normal nutrition and low hs-CRP level; normal nutrition and high hs-CRP level; malnutrition and low hs-CRP level; and malnutrition and high hs-CRP level) were 7.29, 7.12, 10.71, and 17.31%, respectively. Compared with normal nutrition and low hs-CRP level, an isolated condition of either malnutrition or high hs-CRP level was not associated with any significant risk for all-cause mortality. However, concomitant presence of both high hs-CRP level and malnutrition condition was associated with a significantly increased risk of all-cause mortality (HR: 1.51; 95% CI: 1.20–1.89; *p* < 0.001). The *p*-value for interaction between nutritional status and hs-CRP level on all-cause mortality was 0.03.

**Conclusion:**

The interplay of inflammation and malnutrition in patients with diabetics significantly amplifies the deleterious effects of each as distinct disease entities. A prospective randomized clinical trial is needed in the future to verify the results.

## Introduction

Diabetes mellitus (DM) is a highly prevalent disease and associated with a large cardiovascular disease (CVD) burden (1, 2). The chronic low-grade inflammation, characterized by abnormal cytokine production, increased acute-phase proteins, and activation of inflammatory signaling pathways, plays an important role in the pathophysiology of atherosclerosis and DM (3, 4). Among the numerous inflammatory biomarkers, high-sensitivity C-reactive protein (hs-CRP) has the most extensive predictive validation regard to poor prognosis (5).

As a metabolic disease, malnutrition is also common in DM. The recent evidence has proved that malnutrition is the independent risk factor for poor prognosis in patients with DM (6, 7). The Controlling Nutritional Status (CONUT) score is one of the many tools to assess nutritional status, with easy to use, and has recently been widely used to predict the prognosis of DM, cancer, and acute coronary syndrome (ACS) (8–10). Furthermore, strong links have been shown that existed between malnutrition and inflammation (11). Understanding the potential interplay of malnutrition and inflammation on all-cause mortality in patients with DM may allow more personalized management of patients with high or low risk.

Therefore, this study aimed to explore whether the presence of malnutrition can modify the association between inflammation and all-cause mortality.

## Materials and Methods

### Data Sources and Study Population

**Figure 1:**
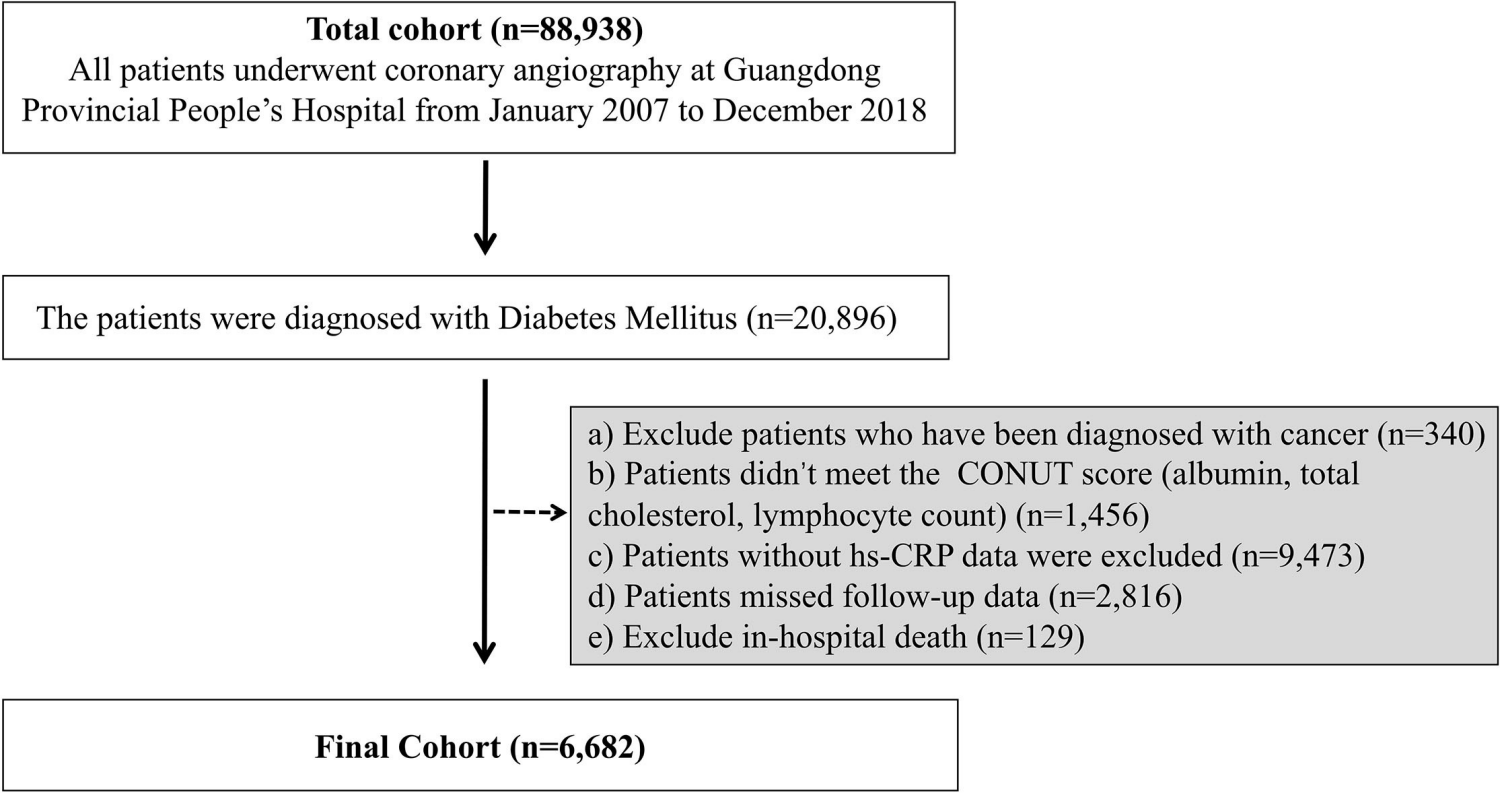
Patient flow diagram.

The Cardiorenal ImprovemeNt (CIN) study is a single-center, retrospective, and observational study, enrolling 88,938 consecutive patients undergoing coronary angiography (CAG) or percutaneous coronary intervention (PCI) in Guangdong Provincial People's Hospital, Guangdong, China, hospitalized in between January 2007 to December 2018 (ClinicalTrials.gov NCT04407936). This study identified the individuals diagnosed with DM (*n* = 20,896). Exclusive criteria included the following: a) Patients were diagnosed with cancer (*n* = 340); b) patients without data of CONUT score (albumin, total cholesterol and lymphocyte count) (*n* = 1,456); c) patients without data of baseline hs-CRP (*n* = 9,473); d) patients without follow-up data (*n* = 2,816); e) patients with in-hospital death (*n* = 129). These data were missing randomly. Eventually, 6,682 patients were included (Figure 1).

First, all traceable personal identifiers were removed from the analytic dataset to protect patients' privacy. Second, the study does not exceed the minimum risk after review by the Guangdong Provincial People's Hospital Ethics Committee. So, the informed consent was not required for this study. The study protocol was approved by Guangdong Provincial People's Hospital ethics committee and the study was performed according to the declaration of Helsinki [No. GDREC2019555H (R1)].

### Baseline Data Collection

From January 2007 to December 2018, the data were extracted from the electronic clinical management records system of the Guangdong Provincial People's Hospital. We had access to all primary and secondary care records. The baseline information included demographic characteristics, coexisting conditions, laboratory examinations, and medications at discharge. The laboratory examinations were measured by an overnight fasting venous blood sample. The death of patients after discharge were recorded by the attending physician or a trained research assistant at the follow-up.

### Endpoint and Clinical Definition

The primary endpoint was all-cause death, which was monitored and recorded by trained nurses and research assistants through outpatient interviews and telephones. The CONUT score takes into account serum albumin, total cholesterol, and lymphocyte count (12). A score of scores of 2–4, 5–8, and 9–12 reflect mild, moderate, and severe malnutrition, respectively (Supplementary Table 1). The malnutrition was defined as CONUT score, which was more than 1. According to nutritional assessment score, patients were divided into two groups or three groups, respectively (CONUT S1, normal; CONUT S2, malnutrition; three groups: CONUT T1, normal; CONUT T2, mild malnutrition; CONUT T3; moderate and severe malnutrition). According to the median hs-CRP level of the study population, the hs-CRP level was dichotomized into two groups [(low hs-CRP level, S1) and (high hs-CRP level, S2)]. The hs-CRP level was trichotomized into three groups [(lowest hs-CRP level, T1), (medium hs-CRP level, T2), (highest hs-CRP level, T3)]. The patients were divided into four groups (normal nutrition and low hs-CRP level *vs*. normal nutrition and high hs-CRP level *vs*. malnutrition and low hs-CRP level *vs*. malnutrition and high hs-CRP level). The estimated glomerular filtration rate (eGFR) was calculated by the Modification of Diet in Renal Disease (MDRD) formula (13). Congestive heart failure (CHF) was defined as New York Heart Association (NYHA) class >2 or Killip class >1 (14). Anemia was defined as a hematocrit ≤39% (male) or ≤36% (female) (15). The DM was defined using International Classification of Diseases-10 (ICD-10) codes or hypoglycemic medication use. Acute myocardial infarction (AMI) and hypertension were defined using ICD-10 codes.

### Statistical Analysis

Continuous variables with the baseline characteristics conforming to the normal distribution are presented as mean ± SDs, and the others that are not conforming to the normal distribution are presented as quartiles [median (25, 75%)] by Kolmogorov–Smirnov test. In addition, the baseline characteristics are presented as proportions for categorical variables. Since the distribution of hs-CRP levels was skewed to the right, natural log-transformation for CRP (ln hs-CRP) was used to normalize the data for the statistical analysis. The differences of four groups in the baseline characteristics were compared through the use of Chi-square tests for categoric variables and a generalized paired *t*-test for continuous variables. The time-to-event data are presented graphically using Kaplan–Meier curves. Log–rank tests were used to compare survival between groups. The multivariate Cox proportional hazards analysis was constructed to examine the association among inflammation, malnutrition, and all-cause mortality adjusted for demographic characteristics, medical history, laboratory tests, and medications. The *p*-value for interactions between categories of hs-CRP and nutritional status for association of outcomes were estimated using the Wald Chi-square test. Presented tests were 2-tailed for all, and *p* < 0.05 was considered statistically significant. All statistical analyses were performed using R (version 4.0.3).

## Results

### Baseline Characteristics of Study Population

There were 6,682 patients included in total in this study (mean age: 63.6 ± 10.0 years, 32% were female). Among the patients with both high hs-CRP level and malnutrition, the prevalence of anemia, chronic kidney disease (CKD) and the level of pro-BNP increased in a stepwise manner, while the level of eGFR and left ventricular ejection fraction (LVEF) decreased (Table 1). The patients with high hs-CRP level had higher prevalence of AMI, CHF, PCI, coronary artery disease (CAD), and more likely to have angiotensin-converting enzyme inhibitor or angiotensin receptor blocker, aspirin and beta-blockers listed as a home medication (Supplementary Table 2). The patients with malnutrition were older and more likely to have comorbidities including AMI, CHF, PCI, pre-AMI, stroke and CAD. Those patients were more often prescribed CCB (Supplementary Table 3). More data on the baseline characteristics of study population are detailed in Table 1.

**Table 1:** Baseline characteristics across CONUT score and high-sensitivity C-reactive protein categories.

**Characteristics**		**G1**	**G2**	**G3**	**G4**	***p***	**%, Std.Diff**		
	**Overall**	**Normal nutrition and**	**Normal nutrition and**	**Malnutrition and**	**Malnutrition and**				
		**Low hs-CRP level**	**High hs-CRP level**	**Low hs-CRP level**	**High hs-CRP level**		**G2 *vs*. G1**	**G3 *vs*. G1**	**G4 *vs*. G1**
		***n* = 1,591**	***n* = 1,208**	***n* = 1,746**	***n* = 2,137**				
**Demographic characteristics**									
Age, year	63.6 ± 10.0	62.0 ± 9.4	61.6 ± 10.0	64.9 ± 9.8	64.8 ± 10.2	<0.001	4.0	30.3	29.3
Female, *n* (%)	2137 (31.98)	608 (38.21)	449 (37.17)	480 (27.49)	600 (28.08)	<0.001	2.2	23.0	21.7
**Medical history**									
AMI, *n* (%)	1024 (15.32)	64 (4.02)	218 (18.05)	111 (6.36)	631 (29.53)	<0.001	45.9	10.5	72.6
CHF, *n* (%)	747 (11.18)	92 (5.78)	108 (8.94)	148 (8.48)	399 (18.67)	<0.001	12.1	10.5	40.1
Anemia, *n* (%)	2438 (36.49)	341 (21.43)	297 (24.59)	688 (39.40)	1112 (52.04)	<0.001	7.5	39.8	66.9
Hypertension, *n* (%)	4507 (67.45)	1032 (64.86)	825 (68.29)	1186 (67.93)	1464 (68.51)	0.089	7.3	6.5	7.7
PCI, *n* (%)	4261 (63.77)	931 (58.52)	802 (66.39)	1013 (58.02)	1515 (70.89)	<0.001	16.3	1.0	26.1
CKD, *n* (%)	1526 (22.84)	199 (12.51)	214 (17.72)	383 (21.94)	730 (34.16)	<0.001	14.6	25.2	53.0
preAMI, *n* (%)	366 (5.48)	85 (5.34)	39 (3.23)	124 (7.10)	118 (5.52)	<0.001	10.5	7.3	0.8
stroke, *n* (%)	523 (7.83)	97 (6.10)	84 (6.95)	142 (8.13)	200 (9.36)	0.002	3.5	7.9	12.2
CAD, *n* (%)	5633 (84.30)	1262 (79.32)	1019 (84.35)	1452 (83.16)	1900 (88.91)	<0.001	13.1	9.8	26.5
**Laboratory tests**									
WBC, 10^9^/L	7.92 ± 2.43	7.34 ± 1.73	8.56 ± 2.19	7.10 ± 1.97	8.66 ± 2.95	<0.001	61.9	13.2	54.4
HGB, g/L	130.83 ± 17.53	136.04 ± 15.07	135.55 ± 16.02	129.64 ± 16.94	125.24 ± 18.64	<0.001	3.1	39.9	63.7
Cholesterol, mmol/L	4.43 ± 1.23	4.93 ± 1.01	5.20 ± 1.15	3.74 ± 1.06	4.19 ± 1.15	<0.001	24.7	114.6	68.1
LDL-C, mmol/L	2.73 ± 0.96	3.05 ± 0.83	3.33 ± 0.89	2.19 ± 0.81	2.61 ± 0.91	<0.001	31.7	105.7	51.3
HDL-C, mmol/L	0.95 ± 0.24	1.04 ± 0.25	0.98 ± 0.23	0.94 ± 0.23	0.88 ± 0.24	<0.001	24.9	39.8	63.1
LVEF, %	58.68 ± 12.29	61.72 ± 10.50	60.70 ± 10.81	59.79 ± 11.97	54.30 ± 13.37	<0.001	9.5	17.1	61.7
LYM, 10^9^/L	1.97 ± 0.71	2.26 ± 0.62	2.25 ± 0.62	1.85 ± 0.70	1.71 ± 0.69	<0.001	2.8	63.1	83.8
ALB, g/L	36.54 ± 4.30	39.24 ± 2.80	38.52 ± 2.50	36.71 ± 3.77	33.28 ± 4.30	<0.001	27.2	76.3	164.4
eGFR, mL/min/1.73 m^2^	77.12 ± 27.17	84.16 ± 24.42	80.58 ± 25.31	77.30 ± 26.04	70.33 ± 29.14	<0.001	14.4	27.2	51.5
HbA1c, %	7.74 ± 1.63	7.62 ± 1.55	7.86 ± 1.57	7.53 ± 1.59	7.92 ± 1.71	<0.001	15.4	6.0	18.0
ProBNP, pg/ml	237.40 [63.14, 1029.00]	93.84 [36.44, 331.40]	184.30 [59.94, 655.62]	165.60 [58.28, 719.25]	903.20 [200.20, 3181.00]	<0.001	19.1	23.7	61.1
**Medications**									
ACEI or ARB, *n* (%)	2861 (43.27)	642 (40.50)	577 (48.12)	665 (38.24)	977 (46.77)	<0.001	15.4	4.6	12.7
Beta-blockers, *n* (%)	5267 (79.66)	1235 (77.92)	989 (82.49)	1362 (78.32)	1681 (80.47)	0.009	11.5	1.0	6.3
Statins, *n* (%)	6128 (92.68)	1454 (91.74)	1123 (93.66)	1608 (92.47)	1943 (93.01)	0.236	7.4	2.7	4.8
Aspirin, *n* (%)	5668 (85.72)	1327 (83.72)	1049 (87.49)	1467 (84.36)	1825 (87.36)	0.001	10.7	1.7	10.4
OAD, *n* (%)	4147 (62.72)	1013 (63.91)	796 (66.39)	1079 (62.05)	1259 (60.27)	0.004	5.2	3.9	7.5
CCB, *n* (%)	1678 (25.38)	373 (23.53)	289 (24.10)	484 (27.83)	532 (25.47)	0.024	1.3	9.9	4.5

*G1, normal nutrition and low hs-CRP level; G2, normal nutrition and high hs-CRP level; G3, malnutrition and low hs-CRP level; G4, malnutrition and high hs-CRP level*.

*hs-CRP, high-sensitivity C-reactive protein; AMI, acute myocardial infarction; CHF, congestive heart failure; PCI, percutaneous coronary intervention; CKD, chronic kidney disease; CAD, coronary artery disease; WBC, white blood cell; HGB, hemoglobin; TRIG, triglyceride; LDL-C, low-density lipoprotein cholesterol; HDL-C, high-density lipoprotein cholesterol; LVEF, left ventricular ejection fraction; LYM, lymphocyte; ALB, albumin; eGFR, estimated glomerular filtration rate; HbA1c, glycosylated hemoglobin; Pro-BNP, pro-brain natriuretic peptide; ACEI or ARB, angiotensin-converting enzyme inhibitor or angiotensin receptor blocker; OAD, oral antidiabetics; CCB, calcium channel blockers*.

### Inflammation, Malnutrition, and All-Cause Mortality

**Figure 2:**
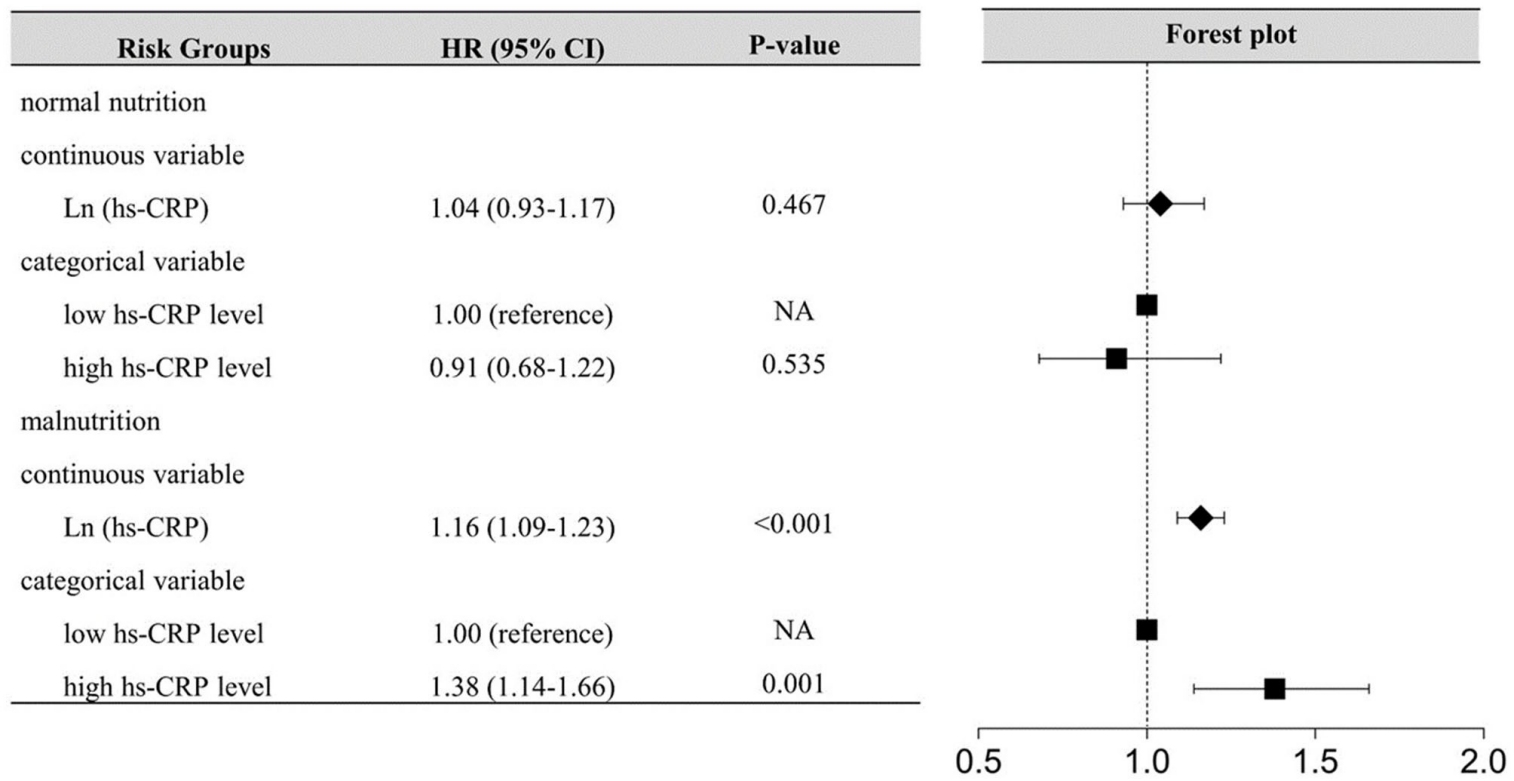
Inflammation-Associated All-cause Mortality Risk According to nutritional status. hs-CRP, High-sensitivity C-reactive protein; The model for inflammation-associated risk according to nutritional status adjusted for age, gender, previous AMI, PCI, anemia, stroke, CHF, CKD, aspirin, AMICAD, angiotensin-converting enzyme inhibitor or angiotensin receptor blocker, beta-blockers, calcium channel blockers, and oral antidiabetics.

During a median follow-up of 5.0 years (interquartile range: 3.0 to 7.6 years), 759 (11.36%) patients died. The high hs-CRP level or malnutrition were associated with increased risk of all-cause mortality in patients with DM (Table 2). In the patients with malnutrition, elevated hs-CRP was strongly associated with increased risk of all-cause mortality, either by continuous [hazard ratios (HR) (95% CI): 1.16 (1.09–1.23)] or dichotomy variables [HR (95% CI):1.38 (1.14–1.66)]. While such association was not significant in those with normal nutrition (*p* > 0.05) (Figure 2). Again, in the setting of low hs-CRP level, no significant association between nutritional status and all-cause mortality risk was observed (*p* > 0.05). However, in the setting of high hs-CRP level, a significant risk of all-cause mortality was observed with malnutrition [HR (95% CI):1.66 (1.29–2.14)] (Supplementary Figure 1).

**Table 2:** Inflammation and malnutrition in relation to all-cause mortality, respectively.

		**Unadjusted**		**Adjusted**	
**Risk groups**	**Event, *n* (%)**	**HR (95%CI)**	***p***	**HR (95%CI)**	***p***
**hs-CRP, mg/dL**					
Ln (hs-CRP), per unit		1.21 (1.16–1.27)	<0.001	1.11 (1.05–1.07)	<0.001
**Seconde**					
hs-CRP S1	303 (9.08)	ref		ref	
hs-CRP S2	456 (13.63)	1.50 (1.30–1.73)	<0.001	1.17 (1.00–1.37)	0.047
**Tertile**					
hs-CRP T1	169 (7.59)	ref		ref	
hs-CRP T2	244 (11.15)	1.40 (1.15–1.70)	0.001	1.31 (1.08–1.60)	0.007
hs-CRP T3	346 (15.26)	1.99 (1.66–2.40)	<0.001	1.50 (1.23–1.84)	<0.001
**CONUT score**					
**Seconde**					
CONUT S1	202 (7.22)	ref		ref	
CONUT S2	557 (14.34)	1.89 (1.61–2.22)	<0.001	1.31 (1.10–1.56)	0.002
**Tertile**					
CONUT T1	202 (7.22)	ref		ref	
CONUT T2	371 (12.00)	1.59 (1.34–1.89)	<0.001	1.26 (1.05–1.50)	0.013
CONUT T3	186 (23.48)	3.04 (2.49–3.71)	<0.001	1.61 (1.27–2.05)	<0.001

*hs-CRP, high-sensitivity C-reactive protein; CONUT score, Controlling Nutritional Status score*.

*hs-CRP S1, low hs-CRP level; hs-CRP S2, high hs-CRP level; hs-CRP T1, lowest hs-CRP level; hs-CRP T2, medium hs-CRP level; hs-CRP T3, highest hs-CRP level; CONUT S1, normal; CONUT S2, malnutrition; CONUT T1, normal; CONUT T2, mild malnutrition; CONUT T3; moderate and severe malnutrition; The hs-CRP model adjusted for age, gender, previous AMI, PCI, anemia, stroke, CHF, CKD, aspirin, AMI, CAD, angiotensin-converting enzyme inhibitor or angiotensin receptor blocker, beta-blockers, calcium channel blockers, CONUT score, and oral antidiabetics*.

*The CONUT model adjusted for age, gender, previous AMI, PCI, anemia, stroke, CHF, CKD, aspirin, AMI, CAD, angiotensin-converting enzyme inhibitor or angiotensin receptor blocker, beta-blockers; calcium channel blockers, hs-CRP, and oral antidiabetics*.

**Figure 3:**
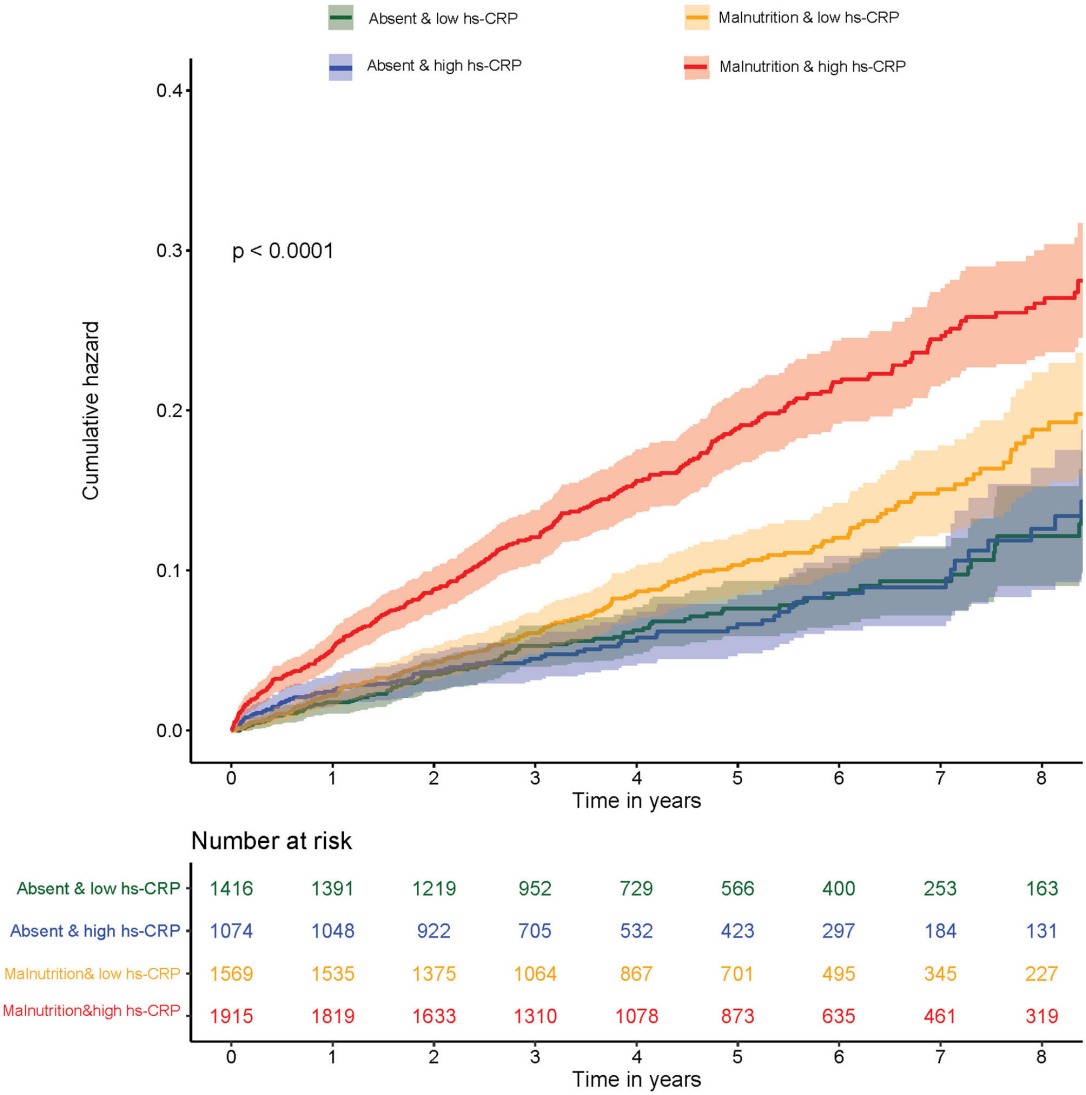
Kaplan–Meier curves of cumulative hazard for all-cause mortality by inflammation and malnutrition.

The mortality of the four groups were 7.29, 7.12, 10.71, and 17.31%, respectively. Kaplan–Meier curves of the cumulative hazard of all-cause mortality among four groups was shown in Figure 3. After controlling for confounding variables, compared with normal nutrition and low hs-CRP level, an isolated condition of either malnutrition or high hs-CRP level was not associated with a significant risk of all-cause mortality. However, concomitant presence of both high hs-CRP level and malnutrition condition were associated with a significantly increased risk of all-cause mortality (HR: 1.51; 95% CI: 1.20–1.89; *p* < 0.001). The *p*-value for interaction between nutritional status (normal nutrition *vs*. malnutrition) and hs-CRP (low hs-CRP level *vs*. high hs-CRP level) on all-cause mortality was 0.03 (Table 3). More information on confounding variables can be detailed in Supplementary Table S4.

**Table 3:** HR of all-cause mortality by inflammation combined with nutritional status.

	**Event, *n* (%)**	**Unadjusted**		**Adjusted**	
**Risk groups**		**HR (95%CI)**	***p***	**HR (95%CI)**	***p***
Normal nutrition & low hs-CRP level	116 (7.29)	ref		ref	
Normal nutrition & high hs-CRP level	86 (7.12)	0.98 (0.74–1.30)	0.903	0.94 (0.71–1.24)	0.653
Malnutrition & low hs-CRP level	187 (10.71)	1.40 (1.11–1.76)	0.005	1.10 (0.87–1.40)	0.425
Malnutrition & high hs-CRP level	370 (17.31)	2.27 (1.84–2.79)	<0.001	1.51 (1.20–1.89)	<0.001
P for interaction		0.003		0.03	

*hs-CRP, high-sensitivity C-reactive protein*.

*p-value for interaction test: 2-way interaction of hs-CRP (low hs-CRP level vs. high hs-CRP level) and nutritional status (normal nutrition vs. malnutrition)*.

*Adjusted for age, gender, previous AMI, PCI, anemia, stroke, CHF, CKD, aspirin, AMI, CAD, angiotensin-converting enzyme inhibitor or angiotensin receptor blocker, beta-blockers, calcium channel blockers, and oral antidiabetics*.

## Discussions

To our knowledge, this is the first study to investigate potential synergistic effects between inflammation and malnutrition in predicting mortality in patients with DM. Several key features of the CIN study, including a large sample size and long follow-up duration allow this question to be addressed in a robust fashion. Our main finding is that high hs-CRP level combined with malnutrition is significantly associated with an increased risk of all-cause mortality among the patients with diabetics, but an isolated condition of either malnutrition or high hs-CRP level was not significantly associated with poor prognosis among those patients.

The first interesting observation extracted from our data is that patients with a higher hs-CRP level had a higher mortality in our cohort. In the study of Gijs et al., the increased CRP were independently associated with cardiovascular and all-cause mortality in primary care treated patients with type 2 diabetes (16). Minna et al. showed that high hs-CRP level had an increased relative risk for CAD death in the cohort of 1,045 patients with diabetes (17). Xin Qian et al. also demonstrated that Serum hs-CRP was predictive of 10-year all-cause death in Chinese adults with hyperglycemia (18). The results of those prior studies were consistent with our research that the mortality is increased in patients with elevated CRP levels among the patients with DM. The inflammation is currently considered as one of the key factors in the pathogenesis of atherosclerosis and is present starting from the earliest stages of the pathology initiation, leading to poor prognosis among patients with diabetics (19).

Diabetes and malnutrition commonly overlap. Our study found that malnutrition was associated with increased risk of all-cause mortality. The finding is not absolutely new, but it was supported in part by a few studies performed in recent years. Gong-Xiang et al. found that malnutrition-assessed by the mini nutritional assessment (MNA) was independently associated with an increased risk of death in 302 elderly patients with diabetes (6). Alejandro et al. also showed a relationship between malnutrition and dying in hospital using the mini nutritional assessment-short form (MNA-SF) as initial screening in 1,014 patients with geriatric diabetics (20). However, those studies conducted so far have been small and may not have been epidemiologically representative of the general population with diabetes.

The most important finding in our study was the patients with diabetics with high hs-CRP level and malnutrition had a significantly increased risk of all-cause mortality. A significant interaction was observed between hs-CRP and nutritional status on all-cause mortality in patients with DM. On the one hand, elevated hs-CRP was strongly associated with increased risk of all-cause mortality, either by continuous or dichotomy variables in patients with malnutrition, while such association was not significant in those with normal nutrition. On the other hand, malnutrition is only associated with increased risk for poor prognosis in patients with diabetics with high hs-CRP level. Compared with those patients with normal nutrition and low hs-CRP level, concomitant condition of both high hs-CRP level and malnutrition was associated with a significantly higher risk of all-cause mortality while the presence of either high hs-CRP level or malnutrition was not significantly associated with an increased risk of all-cause mortality. It should be noted that the patients with high hs-CRP level and malnutrition were more likely to have a higher proportion of major comorbidities (AMI, CKD, and CHF), which indicates that the comorbidities may together constitute risk factors for poor prognosis to a certain extent in the patients with high hs-CRP level and malnutrition. Jagadeswaran et al. demonstrated that malnutrition–inflammation complex syndrome is an important factor that determines mortality in patients with pre-dialysis CKD (21). Rambod et al. also indicated that the increased malnutrition–inflammation score (MIS) is associated with increasing mortality in patients with hemodialysis (22). Given here are the studies that discussed the impact of combined malnutrition and inflammation on the prognosis of different stages of patients with CKD, lacking an exploration of the intrinsic link between the two condition and prognosis. Our research further studied the synergistic effects between hs-CRP and nutritional status and their joint association with all-cause mortality among patients with diabetics. The possible underlying mechanism linking malnutrition and inflammation has not been scientifically detailly described. Dietary protein requirements may be higher in patients with type 2 diabetes to offset reduction in net balance (23), for the restriction carbohydrate intake. This process may allow malnutrition progress further and even develop into hypoalbuminemia which may be correlated to gastrointestinal tract edema (24). It seems that breakdown of the gastrointestinal barrier due to track edema might increase the transfer of bacterial and toxin; thus, aggravating the inflammatory response of the body (25, 26). Moreover, chronic inflammation of the gastrointestinal tract results in the destruction of the intestinal barrier (27), which may enhance the protein catabolism and oxidative stress, leading to anorexia and finally develop into malnutrition (28, 29). The inflammatory state aggravates malnutrition, which in turn can affect the inflammation; thus, forming a vicious circle accelerating the poor prognosis.

Our findings strongly support the need for physicians to practice early assessment, clinical interventions by inflammatory and nutritional status in the patients with diabetics. Malnutrition is often associated with poor prognosis but overlooked in clinical practice. Thus, shifting diet therapy strategies from the treatment of obesity syndrome to prevent malnutrition may be necessary in patients with diabetes. Clinicians should stay abreast of the current scientific evidence to provide meaningful and effective nutritional guidance. Nutritional support knowledge in clinicians is essential to administer DM effectively and to optimize clinical response. Currently, a study reveals physical activity reduces total mortality in the patients with type 2 diabetics with elevated hs-CRP levels. This suggests that the anti-inflammatory effect of physical activity may counteract increased mortality associated with high inflammation (30). The issue of nutrition services in primary health care for patients with diabetics is the first and the only solution for all countries and societies, consisting of optimal energy intake, high intake of protein, vegetables, fish, vitamin D and ω-3 fatty acids, low intake of meat, refined grains, sugar and snacks, and so on (31). Once the patients with diabetics who combined with malnutrition and high inflammation risk have been identified, clinicians should focus on the patient's both conditions, upon hospital admission and follow-up routinely even post-discharge, then adjusting treatment strategies timely. Well-designed and well-executed future studies are needed to explore whether individualized treatments for patients with malnutrition and inflammation will have greater prognostic benefits.

### Study Limitations

Limitations of this study should be considered. Because it was a single-center, retrospective study, our inferences did not reflect direct causality. Unfortunately, information on hematological affection, educational attainment, marital status, socioeconomic characteristics, height, and weight were not available that might help us understand the contributing causes of malnutrition. Due to the lack of other endpoint events, this will limit to generalize our results. In addition, we only included Chinese individuals who undergoing CAG that might be restrictions regarding generalizing to general population; We did not re-evaluate the time-dependent changes in nutritional and inflammatory status.

## Conclusions

The interplay of inflammation and malnutrition in patients with diabetics significantly amplifies the deleterious effects of each as distinct disease entities, and thus may merit closer surveillance and more aggressive management regarding the inflammatory and nutritional status in patients with diabetics. Although we established that hs-CRP might help classify risk in different nutritional status, due to the observational nature of our study, further large-scale clinical studies may be required to verify our results.

## Data Availability Statement

The original contributions presented in the study are included in the article/Supplementary Material, further inquiries can be directed to the corresponding author/s.

## Author Contributions

The authors' responsibilities were as follows—JW, LC, and ZH: research idea and study design. JL, YY, JT, YP, KB, WC, YL, and JX: data acquisition. JL, YY, and XZ: data analysis/interpretation. LC, JL, and ZH: statistical analysis. LC and SC: supervision and mentorship. KC: writing guidance. Each author contributed important intellectual content during manuscript drafting or revision and accepts accountability for the overall work by ensuring that questions on the accuracy or integrity of any portion of the work are appropriately investigated and resolved. All authors read and approved the final version.

## Funding

This research was funded and supported by Fujian Province Natural Science Foundation (Grant number: 2019J01617), Beijing Lisheng Cardiovascular Health Foundation (No. LHJJ20141751), Study on the function and mechanism of the potential target for early warning of cardiorenal syndrome after acute myocardial infarction based on transformism (DFJH201919), Natural Science Foundation of Guangdong Province General Project (2020A1515010940), and Guangdong Provincial Key Laboratory of Coronary Heart Disease Prevention (2017B030314041). The funders had no role in the study design, data collection, and analysis, decision to publish, or preparation of the manuscript; the work was not funded by any industry sponsors.

## Conflict of Interest

The authors declare that the research was conducted in the absence of any commercial or financial relationships that could be construed as a potential conflict of interest.

## Publisher's Note

All claims expressed in this article are solely those of the authors and do not necessarily represent those of their affiliated organizations, or those of the publisher, the editors and the reviewers. Any product that may be evaluated in this article, or claim that may be made by its manufacturer, is not guaranteed or endorsed by the publisher.

## Abbreviations

CAG: coronary angiography
PCI: percutaneous coronary intervention
CONUT: Controlling Nutritional Status
hs-CRP: high-sensitivity C-reactive protein
HR: hazard ratios
DM: diabetes mellitus
ACS: acute coronary syndrome
CVD: cardiovascular disease
CIN: Cardiorenal Improvement Registry
eGFR: estimated glomerular filtration ratel
MDRD: Modification of Diet in Renal Disease
CHF: congestive heart failure
NYHA: New York Heart Association
AMI: acute myocardial infarction
CKD: chronic kidney disease
MIS: malnutrition–inflammation score
MNA-SF: Mini Nutritional Assessment-Short Form.

## References

[1] Dal Canto E Ceriello A Ryden L Ferrini M Hansen TB Schnell O. Diabetes as a cardiovascular risk factor: An overview of global trends of macro and micro vascular complications. Eur J Prev Cardiol. (2019) 26(2_suppl):25–32. 10.1177/2047487319878371 31722562

[2] Low Wang CC Hess CN Hiatt WR Goldfine AB. Clinical update: cardiovascular disease in diabetes mellitus: atherosclerotic cardiovascular disease and heart failure in type 2 diabetes mellitus - mechanisms, management, and clinical considerations. Circulation. (2016) 133:2459–502. 10.1161/CIRCULATIONAHA.116.022194 27297342 PMC4910510

[3] Katakami N. Mechanism of development of atherosclerosis and cardiovascular disease in diabetes mellitus. J Atheroscler Thromb. (2018) 25:27–39. 10.5551/jat.RV17014 28966336 PMC5770221

[4] Tay J Goss A Locher J Ard J Gower BA. Physical function and strength in relation to inflammation in older adults with obesity and increased cardiometabolic risk. (2019) 23:949–57. 10.1007/s12603-019-1260-4 31781724 PMC6996491

[5] Ridker PM. From C-reactive protein to Interleukin-6 to Interleukin-1: moving upstream to identify novel targets for atheroprotection. Circ Res. (2016) 118:145–56. 10.1161/CIRCRESAHA.115.306656 26837745 PMC4793711

[6] Liu GX Chen Y Yang YX Yang K Liang J Wang S. Pilot study of the mini nutritional assessment on predicting outcomes in older adults with type 2 diabetes. Geriatr Gerontol Int. (2017) 17:2485–92. 10.1111/ggi.13110 28657169

[7] Sanz Paris A Garcia JM Gomez-Candela C Burgos R Martin A Matia P. Malnutrition prevalence in hospitalized elderly diabetic patients. Nutr Hosp. (2013) 28: 592–9 10.3305/nh.2013.28.3.6472 23848076

[8] Mineoka Y Ishii M Hashimoto Y Nakamura N Fukui M. Malnutrition assessed by controlling nutritional status is correlated to carotid atherosclerosis in patients with type 2 diabetes. Endocr J. (2019) 66:1073–82. 10.1507/endocrj.EJ19-0107 31434817

[9] Kheirouri S Alizadeh M. Prognostic potential of the preoperative controlling nutritional status (CONUT) score in predicting survival of patients with cancer: a systematic review. Adv Nutr. (2021) 12:234–50. 10.1093/advances/nmaa102 32910812 PMC7850023

[10] Takahashi T Watanabe T Otaki Y Kato S Tamura H Nishiyama S. Prognostic significance of the controlling nutritional (CONUT) score in patients with acute coronary syndrome. Heart Vessels. (2021) 36:1109–16. 10.1007/s00380-021-01792-4 33538856

[11] Nakagomi A Kohashi K Morisawa T Kosugi M Endoh I Kusama Y. Nutritional status is associated with inflammation and predicts a poor outcome in patients with chronic heart failure. (2016) 23:713–27. 10.5551/jat.31526 26782970 PMC7399287

[12] de Ignacio. Ulciated with inflammation and predicts a poor outcome in patients with chha A, et al. CONUT: a tool for controlling nutritional status First validation in a hospital population. Nutr Hosp. (2005) 20:38–45. 15762418

[13] Aguiar-Souto P Ferrante G Del Furia F Barlis P Khurana R Di Mario C. Frequency and predictors of contrast-induced nephropathy after angioplasty for chronic total occlusions. Int J Cardiol. (2010) 139:68–74. 10.1016/j.ijcard.2008.10.006 19056138

[14] Mehran R Aymong ED Nikolsky E Lasic Z Iakovou I Fahy M. A simple risk score for prediction of contrast-induced nephropathy after percutaneous coronary intervention: development and initial validation. J Am Coll Cardiol. (2004) 44:1393–9. 10.1016/S0735-1097(04)01445-7 15464318

[15] Serra M Freeman KP Campora C Sacchini F. Establishment of canine hematology reference intervals for the Sysmex XT-2000iV hematology analyzer using a blood donor database. Vet Clin Pathol. (2012) 41:207–15. 10.1111/j.1939-165X.2012.00417.x 22390629

[16] Landman GW Kleefstra N Groenier KH Bakker SJ Groeneveld GH Bilo HJ. Inflammation biomarkers and mortality prediction in patients with type 2 diabetes (ZODIAC-27). Atherosclerosis. (2016) 250:46–51. 10.1016/j.atherosclerosis.2016.04.015 27179179

[17] Soinio M Marniemi J Laakso M Lehto S R inio M. High-sensitivity C-reactive protein and coronary heart disease mortality in patients with type 2 diabetes: a 7-year follow-up study. (2006) 29:329–33. 10.2337/diacare.29.02.06.dc05-1700 16443882

[18] Qian X He S Wang J Gong Q An Y Li H. Prediction of 10-year mortality using hs-CRP in Chinese people with hyperglycemia: findings from the Da Qing diabetes prevention outcomes study. Diabetes Res Clin Pract. (2021) 173:108668. 10.1016/j.diabres.2021.108668 33453295

[19] Poznyak A Grechko AV Poggio P Myasoedova VA Alfieri V Orekhov AN. The diabetes mellitus-atherosclerosis connection: the role of lipid and glucose metabolism and chronic inflammation. Int J Mol Sci. (2020) 21:1835. 10.3390/ijms21051835 32155866 PMC7084712

[20] Sanz-Paris A Gomez-Candela C Martin-Palmero A Garcia-Almeida JM Burgos-Pelaez R Matia-Martin P. Application of the new ESPEN definition of malnutrition in geriatric diabetic patients during hospitalization: a multicentric study. Clin Nutr. (2016) 35:1564–7. 10.1016/j.clnu.2016.02.018 26997334

[21] Jagadeswaran D Indhumathi E Hemamalini AJ Sivakumar V Soundararajan P Jayakumar M. Inflammation and nutritional status assessment by malnutrition inflammation score and its outcome in pre-dialysis chronic kidney disease patients. Clin Nutr. (2019) 38:341–7. 10.1016/j.clnu.2018.01.001 29398341

[22] Rambod M Bross R Zitterkoph J Benner D Pithia J Colman S. Association of malnutrition-inflammation score with quality of life and mortality in hemodialysis patients: a 5-year prospective cohort study. Am J Kidney Dis. (2009) 53:298–309. 10.1053/j.ajkd.2008.09.018 19070949 PMC5500250

[23] Gougeon R Morais JA Chevalier S Pereira S Lamarche M Marliss EB. Determinants of whole-body protein metabolism in subjects with and without type 2 diabetes. Diabetes Care. (2008) 31:128–33. 10.2337/dc07-1268 17921356

[24] Lee H Han D Kim J Jeon Y Sohn J Hahm J. Successful treatment of protein-losing enteropathy induced by intestinal lymphangiectasia in a liver cirrhosis patient with octreotide: a case report. (2004) 19:466–9. 10.3346/jkms.2004.19.3.466 15201518 PMC2816853

[25] Groschwitz KR Hogan SP. Intestinal barrier function: molecular regulation and disease pathogenesis. J Allergy Clin Immunol. (2009) 124:3–20. 10.1016/j.jaci.2009.05.038 19560575 PMC4266989

[26] Rescigno M. The intestinal epithelial barrier in the control of homeostasis and immunity. Trends Immunol. (2011) 32:256–64. 10.1016/j.it.2011.04.003 21565554

[27] Neurath MF. Targeting immune cell circuits and trafficking in inflammatory bowel disease. Nat Immunol. (2019) 20:970–9. 10.1038/s41590-019-0415-0 31235952

[28] Eckart A Struja T Kutz A Baumgartner A Baumgartner T Zurfluh S. Relationship of nutritional status, inflammation, and serum albumin levels during acute illness: a prospective study. Am J Med. (2020) 133:713–22. 10.1016/j.amjmed.2019.10.031 31751531

[29] Abe M Okada K Maruyama T Maruyama N Matsumoto K Soma M. Relationship between erythropoietin responsiveness, insulin resistance, and malnutrition-inflammation-atherosclerosis (MIA) syndrome in hemodialysis patients with diabetes. Int J Artif Organs. (2011) 34:16–25. 10.5301/IJAO.2011.6314 21298620

[30] Vepsalainen T Soinio M Marniemi J Lehto S Juutilainen A Laakso M. Physical activity, high-sensitivity C-reactive protein, and total and cardiovascular disease mortality in type 2 diabetes. Diabetes Care. (2011) 34:1492–6. 10.2337/dc11-0469 21602429 PMC3120189

[31] Tamura Y Omura T Toyoshima K Araki A. Nutrition management in older adults with diabetes: a review on the importance of shifting prevention strategies from metabolic syndrome to frailty. Nutrients. (2020) 12:3367. 10.3390/nu12113367 33139628 PMC7693664

