# Supplementary material for: A Synergistic Association Between Inflammation, Malnutrition, and Mortality in Patients With Diabetics
Source: Front Nutr. 2022 Jun 2;9:872512. doi: 10.3389/fnut.2022.872512 (PMC9201752; doi:10.3389/fnut.2022.872512)
Supplement: Supplementary file 1 [file Data_Sheet_1.docx]

**Supplementary Table 1.** Nutritional Assessment Tool

| Nutritional Indices | Risk of Malnutrition | | | |
| --- | --- | --- | --- | --- |
|  | Absent | Mild | Moderate | Severe |
| Albumin, g/dl | >3.5 | 3.0-3.49 | 2.5-2.9 | ＜2.5 |
| score | 0 | 2 | 4 | 6 |
| Total cholesterol, mmol/l | >1.60 | 1.2-1.59 | 0.80-1.19 | ＜0.80 |
| score | 0 | 1 | 2 | 3 |
| Lymphocyte count, x109/l | >180 | 140-180 | 100-139 | ＜100 |
| score | 0 | 1 | 2 | 3 |
| Total score | 0-1 | 2-4 | 5-8 | 9-12 |

**Supplementary Table 2.** Baseline characteristics across high-sensitivity reactive protein categories

| **Characteristics** | **Low hs-CRP level** | **High hs-CRP level** | **P-value** |
| --- | --- | --- | --- |
|  | n=3,337 | n=3,345 |  |
| **Demographic characteristics** | | |  |
| Age, year | 63.5 ±9.7 | 63.7 ±10.3 | 0.479 |
| Female, n (%) | 1088 (32.60) | 1049 (31.36) | 0.287 |
| **Medical history** | | |  |
| AMI, n (%) | 175 (5.24) | 849 (25.38) | <0.001 |
| CHF, n (%) | 240 (7.19) | 507 (15.16) | <0.001 |
| Anemia, n (%) | 1029 (30.84) | 1409 (42.12) | <0.001 |
| Hypertension, n (%) | 2218 (66.47) | 2289 (68.43) | 0.092 |
| PCI, n (%) | 1944 (58.26) | 2317 (69.27) | <0.001 |
| CKD, n (%) | 582 (17.44) | 944 (28.22) | <0.001 |
| preAMI, n (%) | 209 (6.26) | 157 (4.69) | 0.006 |
| stroke, n (%) | 239 (7.16) | 284 (8.49) | 0.048 |
| CAD, n (%) | 2714 (81.33) | 2919 (87.26) | <0.001 |
| **Laboratory tests** | | |  |
| WBC, 10^9^/L | 7.21 ±1.86 | 8.62 ±2.70 | <0.001 |
| HGB, g/L | 132.69 ±16.39 | 128.96 ±18.41 | <0.001 |
| TRIG, mmol/L | 1.83 ±1.46 | 1.88 ±1.49 | 0.244 |
| Cholesterol, mmol/L | 4.31 ±1.19 | 4.56 ±1.24 | <0.001 |
| LDL-C, mmol/L | 2.60 ±0.93 | 2.87 ±0.97 | <0.001 |
| HDL-C, mmol/L | 0.99 ±0.24 | 0.92 ±0.24 | <0.001 |
| LVEF, % | 60.73 ±11.31 | 56.63 ±12.87 | <0.001 |
| LYM, 10^9^/L | 2.05 ±0.70 | 1.90 ±0.72 | <0.001 |
| ALB, g/L | 37.92 ±3.57 | 35.17 ±4.52 | <0.001 |
| eGFR, mL/min/1.73m² | 80.51 ±25.52 | 73.88 ±28.29 | <0.001 |
| HbA1c, % | 7.57 ±1.57 | 7.90 ±1.67 | <0.001 |
| ProBNP, pg/ml | 125.90 [45.21, 506.02] | 502.50 [107.00, 1991.00] | <0.001 |
| **Medications** | | |  |
| ACEI or ARB, n (%) | 1307 (39.32) | 1554 (47.26) | <0.001 |
| Beta-blockers, n (%) | 2597 (78.13) | 2670 (81.20) | 0.002 |
| Statins, n (%) | 3062 (92.12) | 3066 (93.25) | 0.086 |
| Aspirin, n (%) | 2794 (84.06) | 2874 (87.41) | <0.001 |
| OAD, n (%) | 2092 (62.94) | 2055 (62.50) | 0.733 |
| CCB, n (%) | 857 (25.78) | 821 (24.97) | 0.465 |

**Abbreviations:** hs-CRP, High-sensitivity C-reactive protein; AMI, acute myocardial infarction; CHF, congestive heart failure; PCI, percutaneous coronary intervention; CKD, chronic kidney disease; CAD, coronary artery disease; WBC, white blood cell; HGB; hemoglobin; TRIG, triglyceride; LDL-C, low-density lipoprotein cholesterol; HDL-C, high-density lipoprotein cholesterol; LVEF; Left Ventricular Ejection Fraction; LYM, lymphocyte; ALB, albumin; eGFR, estimated glomerular filtration rate; HbA1c, glycosylated hemoglobin; Pro-BNP, pro-brain natriuretic peptide; ACEI or ARB, angiotensin-converting enzyme inhibitor or angiotensin receptor blocker; OAD, oral antidiabetics; CCB, calcium channel blockers.

**Supplementary Table 3.** Baseline characteristics across CONUT score categories

| **Characteristics** | **Normal nutrition** | **Malnutrition** | **P-value** |
| --- | --- | --- | --- |
|  | n=2799 | n=3883 |  |
| **Demographic characteristics** | | |  |
| Age, year | 61.8±9.7 | 64.9±10.0 | <0.001 |
| Female, n (%) | 1057 (37.76) | 1080 (27.81) | <0.001 |
| **Medical history** | | |  |
| AMI, n (%) | 282 (10.08) | 742 (19.11) | <0.001 |
| CHF, n (%) | 200 (7.15) | 547 (14.09) | <0.001 |
| Anemia, n (%) | 638 (22.79) | 1800 (46.36) | <0.001 |
| Hypertension, n (%) | 1857 (66.35) | 2650 (68.25) | 0.107 |
| PCI, n (%) | 1733 (61.91) | 2528 (65.10) | 0.008 |
| CKD, n (%) | 413 (14.76) | 1113 (28.66) | <0.001 |
| preAMI, n (%) | 124 (4.43) | 242 (6.23) | 0.002 |
| stroke, n (%) | 181 (6.47) | 342 (8.81) | 0.001 |
| CAD, n (%) | 2281 (81.49) | 3352 (86.33) | <0.001 |
| **Laboratory tests** | | |  |
| WBC, 10^9^/L | 7.87 ±2.03 | 7.95 ±2.67 | 0.144 |
| HGB, g/L | 135.83 ±15.49 | 127.22 ±18.03 | <0.001 |
| TRIG, mmol/L | 2.23 ±1.85 | 1.58 ±1.05 | <0.001 |
| Cholesterol, mmol/L | 5.04 ±1.08 | 3.99 ±1.13 | <0.001 |
| LDL-C, mmol/L | 3.17 ±0.87 | 2.42 ±0.89 | <0.001 |
| HDL-C, mmol/L | 1.01 ±0.24 | 0.91 ±0.24 | <0.001 |
| LVEF, % | 61.28 ±10.64 | 56.75 ±13.05 | <0.001 |
| LYM, 10^9^/L | 2.26 ±0.62 | 1.77 ±0.70 | <0.001 |
| ALB, g/L | 38.93 ±2.70 | 34.82 ±4.41 | <0.001 |
| eGFR, mL/min/1.73m² | 82.60 ±24.87 | 73.39 ±28.03 | <0.001 |
| HbA1c, % | 7.73 ±1.56 | 7.75 ±1.67 | 0.648 |
| ProBNP, pg/ml | 120.00 [44.76, 475.55] | 414.75 [95.75, 1832.50] | <0.001 |
| **Medications** | | |  |
| ACEI or ARB, n (%) | 1219 (43.79) | 1642 (42.89) | 0.486 |
| Beta-blockers, n (%) | 2224 (79.89) | 3043 (79.49) | 0.719 |
| Statins, n (%) | 2577 (92.56) | 3551 (92.76) | 0.795 |
| Aspirin, n (%) | 2376 (85.34) | 3292 (86.00) | 0.475 |
| OAD, n (%) | 1809 (64.98) | 2338 (61.08) | 0.001 |
| CCB, n (%) | 662 (23.78) | 1016 (26.54) | 0.012 |

**Abbreviations:** AMI, acute myocardial infarction; CHF, congestive heart failure; PCI, percutaneous coronary intervention; CKD, chronic kidney disease; CAD, coronary artery disease; WBC, white blood cell; HGB; hemoglobin; TRIG, triglyceride; LDL-C, low-density lipoprotein cholesterol; HDL-C, high-density lipoprotein cholesterol; LVEF; Left Ventricular Ejection Fraction; LYM, lymphocyte; ALB, albumin; eGFR, estimated glomerular filtration rate; HbA1c, glycosylated hemoglobin; Pro-BNP, pro-brain natriuretic peptide; ACEI or ARB, angiotensin-converting enzyme inhibitor or angiotensin receptor blocker; OAD, oral antidiabetics; CCB, calcium channel blockers.

**Supplementary Table 4.** The confounding variables in multivariate Cox proportional hazards analysis

| **Variables** | **Adjusted HR (95%CI)** | **P-value** |
| --- | --- | --- |
| Normal nutrition & low hs-CRP level | ref |  |
| Normal nutrition & high hs-CRP level | 0.94 (0.71-1.24) | 0.653 |
| Malnutrition & low hs-CRP level | 1.10 (0.87-1.40) | 0.425 |
| Malnutrition & high hs-CRP level | 1.51 (1.20-1.89) | <0.001 |
| Age, year | 1.03 (1.02-1.03) | <0.001 |
| gender | 0.87 (0.74-1.02) | 0.096 |
| preAMI | 1.03 (0.77-1.39) | 0.824 |
| PCI | 0.96 (0.79-1.16) | 0.643 |
| Anemia | 1.31 (1.12-1.53) | 0.001 |
| Stroke | 1.23 (0.96-1.56) | 0.102 |
| CHF | 2.23 (1.84-2.71) | <0.001 |
| CKD | 1.57 (1.34-1.86) | <0.001 |
| Aspirin | 0.86 (0.69-1.09) | 0.212 |
| AMI | 0.76 (0.61-0.95) | 0.017 |
| CAD | 1.12 (0.85-1.46) | 0.422 |
| Beta-blockers | 1.21 (0.99-1.48) | 0.058 |
| ACEI or ARB | 1.01 (0.87-1.18) | 0.904 |
| CCB | 1.01 (0.86-1.19) | 0.885 |
| OAD | 0.93 (0.80-1.07) | 0.310 |

**Abbreviations:** pre-AMI, previous acute myocardial infarction; PCI, percutaneous coronary intervention; CHF, congestive heart failure; CKD, chronic kidney disease; AMI, acute myocardial infarction; CAD, coronary artery disease; ACEI or ARB, angiotensin-converting enzyme inhibitor or angiotensin receptor blocker; CCB, calcium channel blockers; OAD, oral antidiabetics.

**Supplementary Figure 1.** Malnutrition-Associated All-cause Mortality Risk According to hs-CRP level


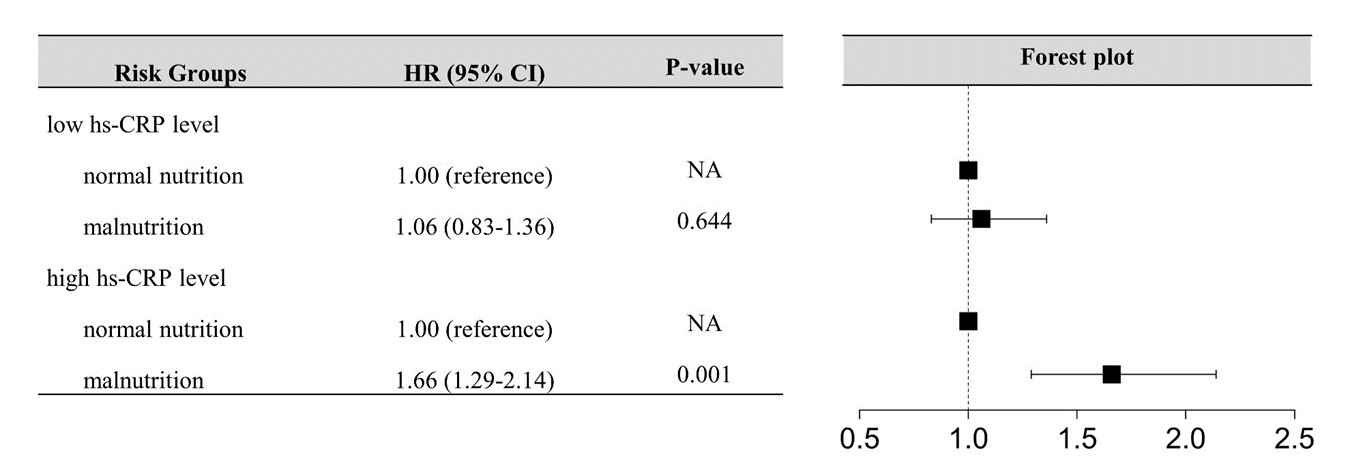


**Note:** hs-CRP, High-sensitivity C-reactive protein;

The model for malnutrition-associated risk according to hs-CRP level adjusted for age, gender, previous acute myocardial infarction, percutaneous coronary intervention, anemia, stroke, congestive heart failure, chronic kidney disease, aspirin, acute myocardial infarction, coronary artery disease, angiotensin-converting enzyme inhibitor or angiotensin receptor blocker, beta-blockers, calcium channel blockers and oral antidiabetics.
